# Supplementary material for: Paleo-proteomic analysis of Iron Age dental calculus provides direct evidence of Scythian reliance on ruminant dairy
Source: PLoS One. 2026 Jan 21;21(1):e0339464. doi: 10.1371/journal.pone.0339464 (PMC12822928; doi:10.1371/journal.pone.0339464)
Supplement: S1 File — (DOCX) [file pone.0339464.s001.docx]

**Paleo-proteomic analysis of Iron Age dental calculus provides direct evidence of Scythian reliance on ruminant dairy**

Jaruschka Pecnik^1,2^*, Alicia R. Ventresca Miller^3,4,5^, Christian Panse^6,7^, Laura Kunz^6^, Antje Dittmann^6^, James A. Johnson^8^, Sergey Makhortykh^9^, Ludmilla Litvinova^9^, Svetlana Andrukh^10^, Gennady Toschev^10^, Michael Krützen^11^, Verena J. Schuenemann^1,2,11,12,13^, S. Wilkin^1,2,5,14^*

^1^ Institute of Evolutionary Medicine, University of Zurich, Zurich, Switzerland

^2^ Department of Environmental Sciences, University of Basel, Basel, Switzerland

^3^ Department of Anthropology, University of Michigan, Ann Arbor, Michigan, United States

^4^ University of Michigan Museum of Anthropological Archaeology, University of Michigan, Ann Arbor, Michigan, United States

^5^ Department of Archaeology, Max Planck Institute for Geoanthropology, Jena, Germany

^6^ Functional Genomics Centre Zurich (FGCZ), ETH and University of Zurich, Zurich, Switzerland

^7^ Swiss Institute of Bioinformatics (SIB), University of Lausanne, Lausanne, Switzerland

^8^ Department of Anthropology, University of Wyoming, Laramie, Wyoming, United States

^9^ Institute of Archaeology of the National Academy of Sciences Ukraine, Kyiv, Ukraine

^10^ Zaporizhzhya National University, Zaporizhzhya, Ukraine

^11^ Department of Evolutionary Anthropology, University of Zurich, Zurich, Switzerland

^12^ Department of Evolutionary Anthropology, University of Vienna, Vienna, Austria

^13^ Human Evolution and Archaeological Sciences (HEAS), University of Vienna, Vienna, Austria

^14^ Australian Research Centre for Human Evolution, Griffith University, Brisbane, Queensland, Australia

*Corresponding Authors: [jaruschka.pecnik@](mailto:jaruschka.pecnik@)uzh.ch; [shevan.wilkin@iem.uzh.ch](mailto:shevan.wilkin@iem.uzh.ch)


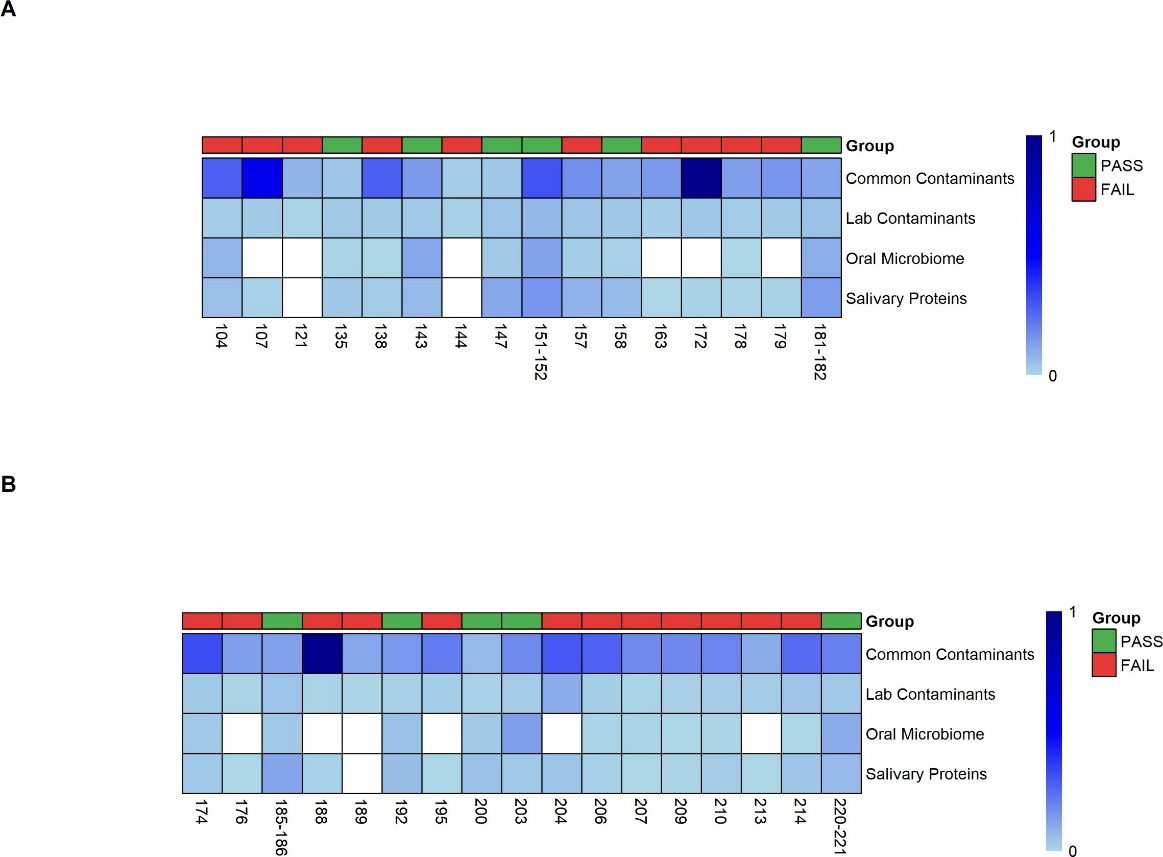


**S1 Fig.** **Oral signature heatmaps of individuals from Bilsk and Mamai-Gora.** The heatmaps represent the peptide-spectrum matches (PSMs) counts in each sample to the four different databases (common and lab contaminants such as oral microbiome and salivary proteins) at Bilsk (A) and Mamai-Gora (B). The group indicates whether the single or merged proteome of each individual passes (PASS) or fails (FAIL) to reach the threshold of 0.5, which signifies an adequate oral signature.


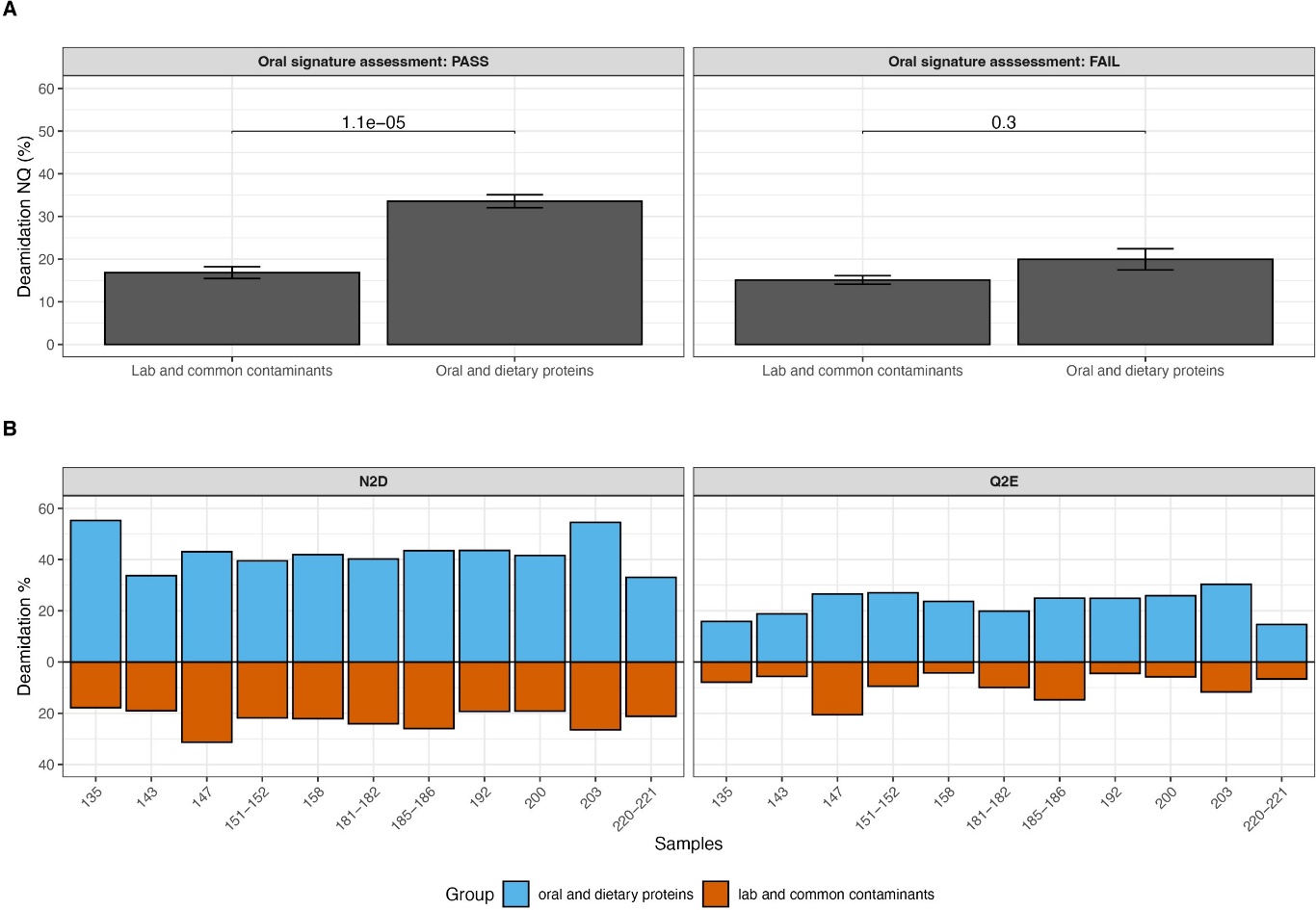


**S2 Fig.** **Bulk deamidation rates of asparagine and glutamine of proteomes that passed and failed the oral signature assessment.** (A) A significant Wilcoxon rank-sum test result was observed only in the proteomes that passed the preservation assessment, indicating that the grouping “oral and dietary proteins” are more deamidated than the “lab and common contaminants”. (B) depicts the deamidation rates of asparagine to aspartic acid (N2D) and of glutamine to glutamic acid (Q2E) for all proteomes that passed the oral signature assessment.
