# Supplementary material for: Paleo-proteomic analysis of Iron Age dental calculus provides direct evidence of Scythian reliance on ruminant dairy
Source: PLoS One. 2026 Jan 21;21(1):e0339464. doi: 10.1371/journal.pone.0339464 (PMC12822928; doi:10.1371/journal.pone.0339464)
Supplement: Attachment — Submitted filename: Response_to_Reviewers_auresp_2.docx [file pone.0339464.s006.docx]

**Response to reviewers**

We would like to express our sincere gratitude to the reviewers for their careful evaluation and valuable insights. We are very thankful that we have the opportunity to submit the following minor revisions of our manuscript titled “Paleo-proteomic analysis of Iron Age dental calculus provides direct evidence of Scythian reliance on ruminant dairy” for the consideration for submission in PLOS One. We have carefully addressed all comments and revised the manuscript accordingly. Page and line numbers refer to the revised manuscript unless otherwise noted.

**Reviewers comments**

**Reviewer #4:**

*I enjoyed reading and assessing this manuscript on the palaeoproteomics of Scythian dental calculus. The topic addressed is important for understanding the occurrence and diversity of dairy use in the Iron Age steppe context. The study is clearly written and appears to be methodologically careful, particularly following the recent manuscript revisions. The identification of BTN1A1 is an interesting and valuable aspect of the work. The reports of horse milk assignment are quite well supported. Overall, this is a well-conducted and interesting contribution, and I have only a few comments that should be straightforward to address.*

*Sample size*

*The sample size is small but carefully filtered, which is entirely appropriate. This approach is preferable to inflating n with data of uncertain or poor quality, and it reflects a commendable level of caution in data selection.*

*Clarification from the response to Reviewer 2*

*In the response to Reviewer 2, the authors state that “the high failure rate is interpreted as reflecting variable preservation rather than laboratory contamination, as no consolidants were used and blanks were clear.” This is a reasonable explanation, but it is not clear whether this statement appears explicitly in the main text. If not, it would be good to include a brief mention of this rationale in the manuscript itself for transparency.*

**Our response:**

Thank you very much for your thoughtful review and kind words. We agree that the manuscript could benefit from our explanation to reviewer #2 from our first revision, thus we have added the following sentence to the results section from line 128 to 131: Since the teeth had not been treated with consolidants and the extraction blanks processed alongside the samples in the dedicated clean laboratory were clean, the high failure rate was interpreted as reflecting variable preservation in the samples.

*Title and framing*

*The title emphasises milk as a staple through the word “reliance.” While this is an engaging framing, it might be a little strong given that the dataset comprises only six samples with milk proteins from two sites. This represents solid direct evidence for dairy consumption, and likely regular consumption, but “reliance” may somewhat overstate the strength of the direct data unless the authors’ argument is that these proteomic findings confirm or complement historical accounts of horse milk use among Scythian groups. Some slight moderation or clarification of this framing could be considered.*

**Our response:**

Thank you very much for your insights. We believe that the title, “Paleo-proteomic analysis of Iron Age dental calculus provides direct evidence of Scythian reliance on ruminant dairy,” accurately reflects the findings of our manuscript, which in our study are interpreted in the context of existing archaeological, historical, and multi-isotopic evidence. Our results show that preserved dietary peptides provide direct evidence for the consumption of ruminant dairy among multiple individuals at both studied sites, whereas horse milk was detected in only one individual. Therefore, the term “reliance” reflects the integration of these proteomic findings with the broader archaeological, historical, and stable isotopic evidence, indicating that ruminant dairy was likely a regular and significant component of their diet. In contrast, the limited horse milk peptide findings do not allow us to make the same claim.

*Threshold at 0.5*

*The choice of a pass threshold at 0.5 “based on inspection” could benefit from a brief justification in the Methods section. For instance, was this threshold selected because it effectively distinguishes between highly contaminated samples and oral-rich proteomes? A short explanatory sentence would help readers understand how this decision was made and improve methodological clarity.*

**Our response:**

Thank you very much for pointing this out. We have adapted to text for further clarification to the following (lines 380 – 381): Based on the visual inspection of each proteome and the comparison of the oral and contaminant PSM counts within the whole dataset, the threshold to pass as an authentic sample was set to 0.5.

*Deamidation*

*The inclusion of deamidation assessment in the revised manuscript is reassuring and significantly strengthens confidence in the authenticity of the results. This is a valuable addition.*

*Protein naming consistency*

*There appear to be inconsistencies in the naming of BTN1A1 / BTNS1A1 / BTS1A1. These should be standardised throughout the manuscript for clarity and accuracy.*

**Our response:**

Thank you very much for noticing the inconsistencies in the naming of Butyrophilin subfamily 1 member A1. We have adapted all of those instances to the correct name of BTN1A1 (i.e. lines: 156, 162, and 221, and Table 1).

*Data availability*

*In the tracked version, the following statement appears:*

*“Mass spectrometry proteomics data have been deposited on MassIVE (massive.ucsd.edu; MSV000092635). For reviewer access use the username: MSV000092635_reviewer and the password: Scythian-Paper!.”*

*This is perfectly acceptable during peer review but would raise a minor concern if carried into the published version, as it suggests the repository may not yet be fully public. PLOS will expect the dataset to be completely open (no login required) upon publication.*

*In the final version, the Data Availability statement should therefore read simply:*

*“Mass spectrometry proteomics data are available on MassIVE (MSV000092635).”*

*No usernames or passwords should be included.*

**Our response:**

Thank you for your comment. We have already made the data public on MassIVE during the last revision process and therefore the sentence with the password credentials was flagged as deleted in the manuscript with track changes document. Nevertheless, we have amended the text according to your suggestion, so it reads clearer.

*Table 1 – Locality of individual (Sr)*

*In Table 1, the column “Locality of individual (Sr)” includes the entry “born outside and moved as a child.” I understand this derives from a previous study, but it remains an interpretation rather than a directly observed result. While it may be a strong inference, it is still an inference. The column heading or note should make clear that this is an interpretive statement rather than a direct measurement.*

**Our response:**

Thank you for your comment. We agree that the column could benefit from clarification. Therefore, we have amended the column title with an asterisk and added an explanation at the bottom of the table (line 149), which reads like this: * Interpretation based on strontium isotopic data (^87^Sr/^86^Sr).

*Overall, this is a thoughtful and well-constructed study that makes a meaningful contribution to the growing field of ancient proteomics. With a few minor clarifications and adjustments as suggested above, it will make a solid addition to the literature.*

**Our response:**

We want to express at this point again great gratitude to the careful review and thoughtful comments of Robert C. Power.
